# A Pentaplex PCR Assay for the Detection and Differentiation of *Shigella* Species

**DOI:** 10.1155/2013/412370

**Published:** 2013-02-13

**Authors:** Suvash Chandra Ojha, Chan Yean Yean, Asma Ismail, Kirnpal-Kaur Banga Singh

**Affiliations:** ^1^Department of Medical Microbiology & Parasitology, School of Medical Sciences, Universiti Sains Malaysia, Health Campus, 16150 Kubang Kerian, Kelantan, Malaysia; ^2^Institute for Research in Molecular Medicine (INFORMM), Universiti Sains Malaysia, Health Campus, 16150 Kubang Kerian, Kelantan, Malaysia

## Abstract

The magnitude of shigellosis in developing countries is largely unknown because an affordable detection method is not available. Current laboratory diagnosis of *Shigella* spp. is laborious and time consuming and has low sensitivity. Hence, in the present study, a molecular-based diagnostic assay which amplifies simultaneously four specific genes to identify *invC* for *Shigella* genus, *rfc* for *S. flexneri*, *wbgZ* for *S. sonnei*, and *rfpB* for *S. dysenteriae*, as well as one internal control (*ompA*) gene, was developed in a single reaction to detect and differentiate *Shigella* spp. Validation with 120 *Shigella* strains and 37 non-*Shigella* strains yielded 100% specificity. The sensitivity of the PCR was 100 pg of genomic DNA, 5.4 × 10^4^ CFU/ml, or approximately 120 CFU per reaction mixture of bacteria. The sensitivity of the pentaplex PCR assay was further improved following preincubation of the stool samples in Gram-negative broth. A preliminary study with 30 diarrhoeal specimens resulted in no cross-reaction with other non-*Shigella* strains tested. We conclude that the developed pentaplex PCR assay is robust and can provide information about the four target genes that are essential for the identification of the *Shigella* genus and the three *Shigella* species responsible for the majority of shigellosis cases.

## 1. Introduction

 Shigellosis continues to be a major health problem in many parts of the world, particularly in underdeveloped and developing countries with poor sanitary systems and improper treatment of water supplies, and also among travelers from industrialized nations [1, 2]. Worldwide, mortality and morbidity due to shigellosis were found to be highest among young children 1 to 5 years of age and the elderly [3–5]. Three species of *Shigella* are responsible for the majority of shigellosis cases: *S. flexneri*, *S. sonnei*, and* S. dysenteriae*. Of these, *S. sonnei* is encountered mostly in industrialized countries and *S. flexneri* in developing countries; *S. dysenteriae* is the only epidemic and pandemic strain [2, 4, 6, 7]. The pathogenesis of shigellosis includes inflammation, ulceration, haemorrhage, tissue destruction, and fibrosis of the colonic mucosa, which result in abdominal pain and diarrhoea/dysentery; in some cases infertility and endometriosis also have been reported [8, 9]. Bacteraemia may occur in people with severe infections, particularly in malnourished children and AIDS patients [10]. A more recent annual estimate of shigellosis throughout the world was estimated to be 90 million incidences and 108,000 deaths [11].

*Shigella *infection spreads by the faecal-oral route. Because of the low infectious dose (10 to 100 organisms), person-to-person transmission is likely the most common route of infection, as the bacteria can survive gastric acidity better than other enteric bacteria [10, 12]. However, transmission via contaminated water, food, overcrowded communities, food handlers, contaminated swimming pools, and flies also has been documented [8, 13, 14]. Recent increases in the number of cases of shigellosis in many parts of the world are attributed to the emergence of multiple-drug resistant strains. Early and accurate diagnosis of shigellosis coupled with prompt medical intervention is essential for reducing the morbidity and mortality caused by *Shigella* spp. 

*Shigella* spp. are fragile organisms that are excreted in large numbers in the stool, but they die off quickly because stools are acidic [15]. Thus, routine microbiological methods used to identify *Shigella* spp. from stool samples are relatively inefficient, time consuming, and labor intensive, and the diagnosis often remains obscure due to the presence of low numbers of causative organisms, competition from other commensal organisms, and inappropriate sample collection. If samples are collected after antibiotic therapy, growth of the organism may be impaired. Moreover, Dutta et al. [16] and Islam et al. [17] reported the sensitivity of the culture method to be 54% and 74%, respectively, compared to that of the conventional PCR technique. Recent molecular diagnostic techniques based on nucleic acids, such as PCR, have shown tremendous potential for identifying *Shigella* spp. and have been increasingly exploited.

 To date, few studies have focused on the rapid diagnosis of shigellosis in underdeveloped and developing countries. However, PCR diagnostic tests have proven to be rapid and effective for the detection and identification to *Shigella* spp. [16–18]. In this study, we searched for genes unique to the *Shigella* serovars and used them to design a pentaplex PCR assay. Our assay differs from conventional multiplex PCRs, which often target the invasion plasmid H (*ipaH*) gene, O antigen synthesis genes, and the 16S rRNA gene for detection of *Shigella* spp. [18–20]; in those cases, the diagnosis is often based on sequence polymorphisms or differences rather than on the absence or presence of a gene. Those methods do not detect *Shigella* at the genus and species level simultaneously. The goal of the present study was to design a pentaplex PCR of *Shigella* spp. with an internal control for the detection of the genus *Shigella *and also for the clinically important *Shigella *spp., namely, *S. flexneri, S. sonnei, *and *S. dysenteriae*. 

## 2. Methods 

### 2.1. Bacterial Strains and Growth Conditions

A total of 120 *Shigella *strains of *S. flexneri* (*n* = 95), *S. sonnei* (*n* = 20), *S. dysenteriae* (*n* = 3) and *S. boydii* (*n* = 2), were used in this study. Pure culture strains were isolated from patients admitted to Hospital Universiti Sains Malaysia (HUSM) from 2001 to 2009.  Table 2 lists the *Shigella *spp. reference strains and other bacteria used in this study. Non-*Shigella* strains were used to determine the specificity and robustness of the assay. All the strains were biochemically and serologically confirmed and were stored at −80°C in 16% glycerol.

### 2.2. Isolation of *Shigella *Spp. from Clinical Specimens Using a Conventional Method

Stool specimens were inoculated on MacConkey (Oxoid Ltd., UK) and deoxycholate citrate agar (DCA) (Oxoid Ltd., UK) using a sterile inoculating loop. Stools were also enriched in selenite F broth (Oxoid Ltd., UK) and incubated overnight at 37 ± 2°C. The next day, the enriched broth was subcultured on MacConkey agar and DCA and incubated overnight at 37 ± 2°C. Colonies morphologically resembling *Shigella* spp. were further evaluated with biochemical tests using triple sugar iron (Oxoid Ltd., UK), urea agar slant (Oxoid Ltd., UK), methyl red (Oxoid Ltd., UK), Simmon's citrate agar slant (Oxoid Ltd., UK), and sulphur indole motility medium (Oxoid Ltd., UK). Identities of colonies were serologically confirmed by slide agglutination with appropriate group-specific polyvalent antisera followed by type-specific monovalent antisera (Denka-Seiken, Tokyo, Japan). Nonserotypable isolates were further checked using an API 20E kit (BioMerieux, Marcy I'Etoile, France). 

### 2.3. Primer Design for Pentaplex PCR Assay

The gene sequence for *invC *of the genus *Shigella* and gene sequences for* rfc, wbgZ*, and* rfpB *of *S. flexneri, S. sonnei*, and* S. dysenteriae*, respectively, were obtained from GenBank [21] for DNA sequence alignment and primer design. The ClustalW program in Vector NTI version 9.0 software (Invitrogen, Carlsbad, CA, USA) was used to align the DNA sequences. The conserved and non-conserved regions of the DNA sequence alignments were visualized using GeneDoc software [22].

**Figure 1:**
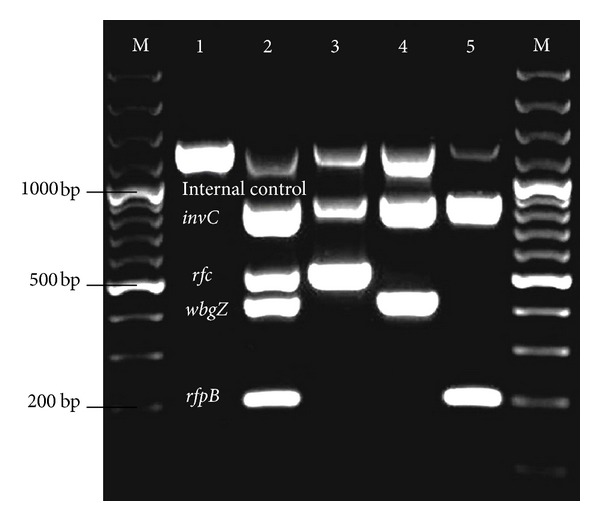
Pentaplex PCR assay profile with reference strains. M, 100 bp plus marker; lane 1, negative control; lane 2, positive control; lane 3, SH052 strain (*rfc S. flexneri*, *invC*-*Shigella* genus); lane 4, SH031 strain (*invC*-*Shigella* genus, *wbgZ  S. sonnei*); lane 5, SD375 strain (*invC*-*Shigella* genus, *rfpB S. dysenteriae*); M, 100 bp plus marker.

Based on the conserved regions of the alignment, specific primer pairs for the genus *Shigella *were designed to amplify the *invC* gene. Specific primers for *S. flexneri*, *S. sonnei*, and *S. dysenteriae* were designed based on the non-conserved regions of *rfc*, *wbgZ*, and *rfpB* genes, respectively. The four primer pairs were designed in such a way that amplification efficiency was not hindered and amplicon sizes ranging from 211 to 875 bp could be differentiated by agarose gel electrophoresis (Figure 1). The homology of the designed primer sequences was analyzed using BLAST [21]. A primer pair based on the *ompA* gene was designed (1319 bp) and used as an internal control. The primer (AIT BIOTECH, Singapore) sequences for the five genes and expected PCR product sizes are shown in Table 1.

### 2.4. Pentaplex PCR Assay

The pentaplex PCR assay was standardized using genomic DNA extracted from reference *Shigella *spp. A mixture of DNA from three strains (*S. flexneri *(SH052), *S. sonnei *(SH023), and *S. dysenteriae *(SD375)) that contained the four genes of interest was used as a positive control. DNase-free distilled water was used as a negative control. In addition, a plasmid containing the *ompA* gene (10 pg) was incorporated as an internal control template to rule out false negative results. An internal control (primer pair and template) was incorporated into every reaction mixture, including negative controls.

 The colonies isolated from blood agar were inoculated into nutrient agar (Oxoid Ltd., UK) and incubated overnight at 37 ± 2°C. Bacteria lysate was prepared by resuspending one bacterial colony in 30 *μ*L of deionized water, boiling for 5 min, and centrifuging at 8000 ×g for 2 min. Two microliters of supernatant then were used as the DNA template in the pentaplex PCR assays.

The optimized primer concentration for each gene (0.4 pmol for *ompA*,* rfc*, and *rfpB*; 0.3 pmol for *invC*; and 0.2 pmol for *wbgZ*) was used in the pentaplex PCR. The other components used in the PCR were 200 *μ*M dNTPs, 2.5 mM MgCl_2_, 1X PCR buffer, and 1 U *Taq *DNA polymerase (Promega, Madison, WI, USA). The PCR was performed using a Mastercycler Gradient (Eppendorf, Hamburg, Germany) with one cycle of initial denaturation at 94°C for 3 min, 30 cycles of denaturation at 94°C for 30 s, annealing for 30 s at 60°C, and extension at 72°C for 30 s, followed by an extra cycle of annealing at 60°C for 30 s and a final extension at 72°C for 3 min. The PCR products were analyzed by electrophoresis on 1.5% agarose gels (Promega) with 10 mg/mL ethidium bromide (Sigma, USA); they were run at 100 V for 60 min. PCR products were visualized under a UV transilluminator and photographed using an image analyzer (ChemiImager 5500; Alpha Innotech, San Leandro, CA, USA). 

### 2.5. Evaluation of Pentaplex PCR Assay Results

Analytical specificity was evaluated using DNA lysate prepared from pure cultures of 120 *Shigella* strains, 10Gram-positive strains, and 27 Gram negative strains. The analytical sensitivity was evaluated using genomic DNA (1 *μ*g to 10 pg) and also 10^8^ to 10^2^ CFU/mL obtained from *Shigella *strains. The diagnostic evaluation of the pentaplex PCR was conducted using 95 *S. flexneri*, 20 *S. sonnei*, 3* S. dysenteriae*,  and 2* S. boydii *strains. The results were compared with those from the conventional culture method, which is considered to be the standard of detection [23].

### 2.6. Faecal Spiking and Sensitivity

The standardized pentaplex PCR assay designed to detect *Shigella* directly from stool was also tested using stool samples spiked with a known amount of *Shigella* based on slight modification of method described by Houng et al. [18]. Stool samples (*n* = 2, children ≤ 5 years old) were collected from the Department of Medical Microbiology and Parasitology, HUSM, Malaysia, and were pretested for the presence of amplifiable *Shigella* DNA by pentaplex PCR and found to be negative. Five grams of stool were weighed and suspended in 45 mL of normal saline (NS) solution, which corresponds to a 10% mixture. The solution was vortexed for 2 min to obtain a homogenous mixture. Insoluble particulate matter was removed by low-speed centrifugation (1000 ×g) for 3 min, and the supernatant was transferred to a fresh tube. Meanwhile, an overnight culture of *Shigella*-specific strains was grown in nutrient broth (NB) (Oxoid Ltd., UK) under shaking condition (200 rpm). The bacterial count was estimated to be 10^8^ CFU and 10-fold diluted with NS. Next, a 500 *μ*L sample of each dilution of bacterial cells was mixed with 500 *μ*L of the faecal suspension in a new tube. Tubes were vortexed, 1 mL of the mixture was transferred to 9 mL of GNB (Merck, Germany), and the mixture was preincubated at 37 ± 237 ± 2°C for up to 6 h without shaking. At time 0, 2, 4, and 6 h after incubation, 200 *μ*L of mixture was placed in a 0.5 mL microcentrifuge tube and centrifuged at 8000 ×g for 3 min. The supernatant was removed, cells were washed using NS, and lysates were prepared by the boiling method. Two microliters of the lysate supernatant were used for pentaplex PCR evaluation.

### 2.7. Screening of Clinical Specimens

Stool samples were collected from patients suspected with acute gastroenteritis or dysentery from Department of Medical Microbiology and Parasitology, USM, Malaysia. Approximately 1 g of each faecal sample from 30 patients suspected of dysentery was transferred to 9 mL of GNB broth corresponding to 10% mixture and preincubated at 37°C ± 2°C for 4 h without shaking. Subsequently, 200 *μ*L of the suspension was taken out and placed in 0.5 mL microcentrifuge tube and centrifuged at 8000 ×g for 3 min. The supernatant was discarded and cells were washed with 200 *μ*L of 0.9% NS. Pellet was resuspended with 30 *μ*L of PCR grade water and boiled for 5 min. Two microlitres of the supernatant containing DNA (lysate) were used for thermostabilized multiplex PCR evaluation. A pure culture of strain and a *Shigella *spiked faecal sample served as positive controls whilst a PCR reaction mixture without bacterial DNA template and an unspiked faecal sample from a healthy individual were incorporated as negative controls.

## 3. Results

We developed a pentaplex PCR assay that simultaneously amplifies four specific genes and one internal control gene in a single reaction; this assay allows detection and differentiation of *Shigella* at the genus and species levels (Table 1). Based on the compatibility of the primers for different genes, the pentaplex PCR was standardized for the *invC* (genus *Shigella*), *rfc* (*S. flexneri*), *wbgZ *(*S. sonnei*), and *rfpB* (*S. dysenteriae*) genes. The fifth primer set (*ompA*) was used for amplification of the internal control to validate the reliability of the assay and to exclude false negative results. Figure 1 shows a representative gel that illustrates differentiation of *Shigella* by genus and species.

All of the primers were positive for the genes targeted by pentaplex PCR but negative for non-*Shigella* strains (Table 2). The optimum concentration of primer needed to amplify uniformly with approximately the same band intensity was 0.4 pmol for *ompA*, *rfc*, and *rfpB*; 0.3 pmol for *invC*; and 0.2 pmol for *wbgZ*. The pentaplex PCR gave the best results when 2.5 mM MgCl_2_, 200 *μ*M dNTPs, and 1 U *Taq* polymerase were used. The optimal annealing temperature was 60°C.

**Figure 2:**
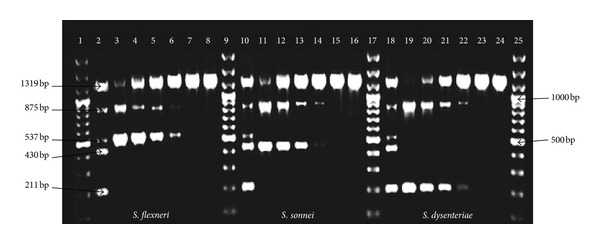
Analytical sensitivity of multiplex PCR at genomic DNA level using reference strains. Lane 1, 100 bp plus marker; lane 2, positive control; lane 3, 100 ng/*μ*L of genomic DNA *S. flexneri*; lane 4, 10 ng/*μ*L of genomic DNA *S. flexneri*; lane 5, 1 ng/*μ*L of genomic DNA *S. flexneri*; lane 6, 100 pg/*μ*L of genomic DNA *S. flexneri*; lane 7, 10 pg/*μ*L of genomic DNA *S. flexneri*; lane 8, 1 pg/*μ*L of genomic DNA *S. flexneri*; lane 9, 100 bp plus marker; lane 10, positive control; lane 11, 100 ng/*μ*L of genomic DNA *S. sonnei*; lane 12, 10 ng/*μ*L of genomic DNA *S. sonnei*; lane 13, 1 ng/*μ*L of genomic DNA *S. sonnei*; lane 14, 100 pg/*μ*L of genomic DNA *S. sonnei*; lane 15, 10 pg/*μ*L of genomic DNA *S. sonnei*; lane 16, 1 pg/*μ*L of genomic DNA *S. sonnei*; lane 17, 100 bp plus marker; lane 18, positive control; lane 19, 100 ng/*μ*L of genomic DNA *S. dysenteriae*; lane 20, 10 ng/*μ*L of genomic DNA *S. dysenteriae*; lane 21, 1 ng/*μ*L of genomic DNA *S. dysenteriae*; lane 22, 100 pg/*μ*L of genomic DNA *S. dysenteriae*; lane 23, 10 pg/*μ*L of genomic DNA *S. dysenteriae*; lane 24, 1 pg/*μ*L of genomic DNA *S. dysenteriae*; lane 25, 100 bp plus marker.

**Figure 3:**
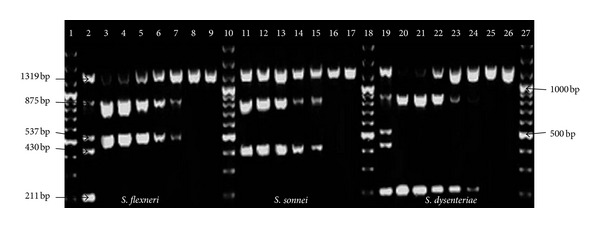
Analytical sensitivity of multiplex PCR at the bacterial level (CFU/mL) using reference strains. Lane 1, 100 bp plus marker; lane 2, positive control; lane 3, 10^8^ CFU/mL lysate of *S. flexneri*; lane 4, 10^7^ CFU/mL lysate of *S. flexneri*; lane 5, 10^6^ CFU/mL lysate of* S. flexneri*; lane 6, 10^5^ CFU/mL lysate of *S. flexneri*; lane 7, 10^4^ CFU/mL lysate of* S. flexneri*; lane 8, 10^3^ CFU/mL lysate of* S. flexneri*; lane 9, 10^2^ CFU/mL lysate of* S. flexneri*; lane 10, 100 bp plus Marker; lane 11, 10^8^ CFU/mL lysate of* S. sonnei*; lane 12, 10^7^ CFU/mL lysate of* S. sonnei*; lane 13, 10^6^ CFU/mL lysate of* S. sonnei*; lane 14, 10^5^ CFU/mL lysate of* S. sonnei*; lane 15, 10^4^ CFU/mL lysate of* S. sonnei*; lane 16, 10^3^ CFU/mL lysate of* S. sonnei*; lane 17, 10^2^ CFU/mL lysate of* S. sonnei*; lane 18, 100 bp plus Marker; lane 19, Positive control; lane 20, 10^8^ CFU/mL lysate of* S. dysenteriae*; lane 21, 10^7^ CFU/mL lysate of* S. dysenteriae*; lane 22, 10^6^ CFU/mL lysate of* S. dysenteriae*; lane 23, 10^5^ CFU/mL lysate of* S. dysenteriae*; lane 24, 10^4^ CFU/mL lysate of* S. dysenteriae*; lane 25, 10^3^ CFU/mL lysate of* S. dysenteriae*; lane 26, 10^2^ CFU/mL lysate of* S. dysenteriae*; lane 27, 100 bp plus marker.

The pentaplex PCR assay was evaluated for analytical specificity and sensitivity. At the DNA level sensitivity was 100 pg of DNA (Figure 2) and at the bacterial level it was 5.4 × 10^4^ CFU/mL or approximately 120 CFU per reaction mixture of bacteria (Figure 3). The analytical specificity of the pentaplex PCR assay was evaluated using 120 clinical strains of *Shigella* spp. (95 *S. flexneri*, 20 *S. sonnei*, 3* S. dysenteriae, *and 2* S. boydii*), 10 Gram positive strains, and 27 Gram negative strains (Table 2). 

Of the 120 *Shigella* strains tested, 116 were positive for *invC*. Of the 20 strains of *S. sonnei*, 16 were positive for *wbgZ*. The fact that four strains were *wbgZ* and *invC* negative suggests that the virulence plasmid might have been lost due to long storage time or subculturing [24]. The *rfc* and *rfpB* primers showed 100% sensitivity in identifying their respective strains (Table 3).

The DNA sequencing results of the PCR amplicons for the four genes were aligned using Vector NTI version 9.0 software and then analyzed by BLAST. The results showed that all four PCR amplicons were specific to their respective genes and had 100% sequence identity with the existing GenBank sequences.

The effect of enrichment for *Shigella* count was investigated by spiking normal stool samples with known *Shigella *numbers and incubating the mixture in growth medium. The sample inoculated with 10^3^ CFU/mL did not generate any amplicon at time zero (before incubation); however *S. flexneri*, *S. sonnei*, and *S. dysenteriae* produced clear amplicons after 4 h of incubation. This result illustrates that it is possible to detect *Shigella* spp. from samples containing low bacterial concentration by preincubating the samples in growth medium. A preliminary study on the efficacy of the multiplex PCR assay was evaluated using 30 faecal samples which were culturally confirmed negative for *Shigella *spp. No target genes were amplified in the multiplex PCR assay although both the positive and internal controls had amplifications. 

## 4. Discussion

Shigellosis is the most communicable of the bacterial diarrhoeas [11]. This disease occurs as sporadic cases and occasional outbreaks of varying magnitude in developed countries and causes epidemics and endemic disease in developing countries. Because shigellosis is highly contagious, it is crucial to develop a rapid method for identifying the bacteria in order to limit and control outbreaks. Classical methods for determining the presence of bacteria in general are time consuming and labor intensive and have low sensitivity [16, 17, 25–27]. Hence, molecular methods, which offer speed, sensitivity, and specificity, have been developed to address this problem. However, some of these methods are relatively expensive and difficult to perform and require special equipment (e.g., a method combining immunocapture with PCR of bacteria for the detection of *Shigella* spp. [28], seminested PCR [29], PCR-nonradioactive labeling [30], PCR-RFLP [31], and PCR-ELISA [32]). On the other hand, DNA microarray analysis proved to be specific, sensitive, and reproducible, but its application as a diagnostic or epidemiological tool is difficult in view of the elevated cost, instruments and requires a skilled person to perform the test [33].

To overcome these drawbacks of existing techniques, we developed a pentaplex PCR assay and evaluated its ability to detect and identify three enteropathogenic bacteria species at the genus and species levels. Several previous studies described the development of *Shigella *multiplex PCR, but those assays did not discriminate between *Shigella* at the genus and species levels, nor did they differentiate *Shigella* from closely related pathogens such as *Salmonella*, *Citrobacter*, and enteroinvasive *Escherichia coli* (EIEC) [20, 25, 34]. 

In our study, primers were designed based on the prevalent species responsible for the majority of shigellosis cases [2, 4, 6, 7]. Four highly specific genes (*invC*, *rfc, wbgZ,* and *rfpB*) that can best detect *Shigella* at the genus and species level were identified. Because *invC *is present among all of the* Shigella *spp., *rfc, wbgZ*, and *rfpB* were combined with *invC* for speciation of the *Shigella* strains. The primer for *S. flexneri* that targets the *rfc* gene was designed based on Houng et al. [18], and it allows discrimination between *Shigella *and EIEC in faecal samples. Similarly, the three other highly specific primers were designed based on the homologous sequences retrieved from GenBank (NCBI). *S. boydii* species identification was not included in this study because of its low prevalence in developing and industrialized countries. However, the presence of the *invC* band specific for *Shigella* genus and the absence of all other amplicons specific for *Shigella* spp. can be considered to be the detection criteria for *S. boydii*. 

Following the successful application of the primers individually, they were mixed to produce the pentaplex PCR. The mixing of primers in a single tube decreases costs and time and increases the ease of the assay. Although numerous reports of PCR assays for the detection of *Shigella* spp. exist [18, 20, 25, 34], only a few of them have incorporated internal controls to rule out false negatives [35]. According to guidelines for Molecular Diagnostic Methods for Infectious Diseases (MM3-A2), incorporation of an internal control in the reaction is essential for the diagnostic test to exclude false negative result or the presence of inhibitors. In the present study, inclusion of a 1319 bp internal control in the pentaplex PCR assay helped us to rule out false negatives or PCR inhibitors. The primers were designed with great care; BLAST and alignment results of the sequence confirmed that it did not cross-react with closely related species such as enteroinvasive *Escherichia coli *(EIEC) which gives rise to similar illness as shigellosis. However, it was unfortunate that EIEC strain was not available to be tested in this study. 

The pentaplex PCR developed in our study successfully amplified all five amplicons from a single reaction tube, and the primers did not interact with each other to produce false negatives. Compatibility of primers with target amplicons was confirmed by sequencing the PCR products derived from the five representative strains. The pentaplex mixture was tested with 120 clinical strains and also against other Gram positive and Gram negative strains to determine the primers' specificity. The primers were found to be highly specific in identifying *Shigella* spp. However, in some cases nonspecific amplicon was weak and fell outside the expected size range for the primers applied and therefore was of no concern. These nonspecific amplifications are likely due to low levels of nonspecific binding between the primers and the bacterial genomic DNA. 

The presence of PCR inhibitors in stool samples (e.g., bilirubin, bile salts, and heme in the faeces) may inhibit amplification and limit the usefulness of PCR technique [36, 37]. As reported by Theron et al. [29] and Thong et al. [20], an enrichment procedure prior to PCR enhances the total number of bacteria present, which helps to dilute the PCR inhibitory substances. As stated by the manufacturer of Gram negative broth (GNB), citrate and deoxycholate in the broth act as selective agents and suppress the growth of Gram positive organisms, including some coliform bacteria. The additional step of preincubating spiked faecal sample in GNB helps to eliminate the natural inhibitors and could enhance the viability of *Shigella* spp. in samples [29, 38]. A preliminary study with clinical specimens showed no cross reaction with other non-*Shigella* strains, however, to check the real performance of the developed test, a larger positive sample size need to be further investigated. The 4 h enrichment step would increase the total number of bacteria present and enhance the sensitivity of the assay. The sensitivity level achieved in our study was comparable to that of other studies. For example, Houng et al. [18] detected up to 7.4 × 10^4^ CFU/mL of *Shigella* by amplifying IS 630 sequences, Yavzori et al. [39] detected at 10^4^ CFU of *Shigella* per gram of faeces with the use of *virF *primers, and Thong et al. [20] reported a detection level of 5.0 × 10^4^ CFU/mL of *Shigella* by amplifying *ial *and *ipaH* sequences in *Shigella* spp. Thus, the average detection of pentaplex PCR described in this study (5.4 × 10^4^ CFU/mL) is within the common detection limit for *Shigella*.

## 5. Conclusion

In conclusion, the pentaplex PCR assay developed in this study was able to detect four genes that are essential for the detection and differentiation of *Shigella* at the genus and species levels simultaneously in a single test within 4 h. The built-in internal control in this assay prevented false negative results. The pentaplex PCR assay was highly sensitive and could provide results on the same day that a specimen was submitted for evaluation, which is critical during outbreaks.

## Figures and Tables

**Table 1:** Sequences of primers used for the pentaplex PCR.

Primers	Primer sequence (5′-3′)	Gene target	Location of gene	Amplicon size (bp)	Target identity	GenBank accession number
SgenDF1	TGC CCA GTT TCT TCA TAC GC	*invC *	Plasmid	875	*Shigella *genus	AF386526
SgenDR1	GAA AGT AGC TCC CGA AAT GC					
SflexDF1	TTT ATG GCT TCT TTG TCG GC	*rfc *	Chromosome	537	*Shigella flexneri *	CP000266
SflexDR1	CTG CGT GAT CCG ACC ATG					
SsonDF1	TCT GAA TAT GCC CTC TAC GCT	*wbgZ *	Plasmid	430	*Shigella sonnei *	CP000039
SsonDR1	GAC AGA GCC CGA AGA ACC G					
SdysDF1	TCT CAA TAA TAG GGA ACA CAG C	*rfpB *	Plasmid	211	*Shigella dysenteriae *	CP000640
SdysDR1	CAT AAA TCA CCA GCA AGG TT					
ICDF1	GCA GGC ATT GCT GGG TAA	*ompA *	Plasmid	1319	Internal control	AY305875
ICDR1	ACA CTT GTA AGT TTT CAA CTA CG					

**Table 2:** Bacterial species and strains used in this study and results of pentaplex PCR.

Bacterial strains	No. of strains tested	*inv C* ^a^	*rfc *	*wbgZ *	*rfpB *	IC (*ompA*)
*S. flexneri* (ATCC 12022)^b^	1	+	+	−	−	+
*S. sonnei* (SH031)^c^	1	+	−	+	−	+
*S. boydii* (ATCC 9207)^b^	1	+	−	−	−	+
*S. dysenteriae* (SD375)^d^	1	+	−	−	+	+
*Salmonella* spp.	2	−	−	−	−	+
*S.* Typhi^c^	3	−	−	−	−	+
*S.* Paratyphi A^c^	1	−	−	−	−	+
*S.* Paratyphi B^c^	1	−	−	−	−	+
*Klebsiella* spp.^c^	2	−	−	−	−	+
*K. p* *ne* *um* *on* *ia* *e* ^c^	2	−	−	−	−	+
*E. coli* (EPEC)^c^	1	−	−	−	−	+
*E. coli* (EHEC)^c^	1	−	−	−	−	+
*E. coli* (ETEC)^c^	1	−	−	−	−	+
*E. c* *ol* *i* ^c^	4	−	−	−	−	+
*V. c* *h* *ol* *e* *ra* *e* ^c^	3	−	−	−	−	+
*V. p* *a* *ra* *h* *em* *ol* *yt* *ic* *us* ^c^	1	−	−	−	−	+
*V. f* *ul* *va* *li* *s* ^c^	1	−	−	−	−	+
*V. cholera* (wild type)^c^	1	−	−	−	−	+
*V. f* *ur* *ni* *ss* *ii* ^c^	1	−	−	−	−	+
*P. a* *er* *ug* *in* *os* *a* ^c^	3	−	−	−	−	+
*P. m* *i* *ra* *b* *i* *li* *s* ^c^	1	−	−	−	−	+
*P. v* *ul* *g* *ar* *is* ^c^	1	−	−	−	−	+
*C. f* *re* *ud* *ii* ^*c*^	1	−	−	−	−	+
*E. c* *lo* *ac* *ae* ^c^	1	−	−	−	−	+
*Y. e* *nt* *er* *oc* *ol* *it* *ic* *a* ^c^	1	−	−	−	−	+
*Acinetobacter* spp.^c^	1	−	−	−	−	+
*A. b* *a* *um* *a* *n* *ni* *i* ^c^	1	−	−	−	−	+
*S. m* *ar* *ce* *s* *ce* *n* *s* ^c^	1	−	−	−	−	+
*Campylobacter* spp.^c^	1	−	−	−	−	+
*A. h* *yd* *ro* *ph* *il* *a* ^c^	1	−	−	−	−	+
*M. m* *or* *ga* *ni* *i* ^c^	1	−	−	−	−	+
*B. c* *er* *e* *us* ^c^	1	−	−	−	−	+
*S. a* *ur* *e* *us* ^c^	2	−	−	−	−	+
Methylene resistant *S. aureus* ^c^	1	−	−	−	−	+
*Streptococcus* spp. Group A^c^	1	−	−	−	−	+
*Streptococcus* spp. Group B^c^	1	−	−	−	−	+
*Streptococcus* spp. Group G^c^	1	−	−	−	−	+
*Corynebacterium* spp.^c^	1	−	−	−	−	+
*Listeria* spp.^c^	1	−	−	−	−	+
*Lactobacillus* spp.^c^	1	−	−	−	−	+
*Gardnerella* spp.^c^	1	−	−	−	−	+

^a^ *Shigella* genus.

^b^Reference strains from American Type Culture Collection (ATCC), Reston, VA, USA.

^c^Department of Medical Microbiology and Parasitology, School of Medical Sciences, Universiti Sains Malaysia.

^d^Obtained from Institute for Medical Research, Malaysia.

“+” is positive; “–” is negative by pentaplex PCR.

**Table 3:** Summary for evaluation of pentaplex PCR assay carried out using reference strains.

Number of strains evaluated by pentaplex PCR assay		
Bacterial strains	No. of specimen tested	Positive (%) (*n* = 120)
*Shigella* genus	120	116 (96.7%)
*S. flexneri *	95	95 (100%)
*S. sonnei *	20	16 (80%)
*S. dysenteriae *	3	3 (100%)
